# Social inequalities in patient-reported outcomes among older multimorbid patients – results of the MultiCare cohort study

**DOI:** 10.1186/s12939-015-0142-6

**Published:** 2015-02-07

**Authors:** Olaf von dem Knesebeck, Horst Bickel, Angela Fuchs, Jochen Gensichen, Susanne Höfels, Steffi G Riedel-Heller, Hans-Helmut König, Karola Mergenthal, Gerhard Schön, Karl Wegscheider, Siegfried Weyerer, Birgitt Wiese, Martin Scherer, Hendrik van den Bussche, Ingmar Schäfer

**Affiliations:** Department of Medical Sociology, University Medical Center Hamburg-Eppendorf, Martinistr. 52, 20246 Hamburg, Germany; Department of Psychiatry, Technical University of Munich, Ismaninger Str. 22, 81675 Munich, Germany; Institute of General Practice, University of Dusseldorf, Moorenstr. 5, 40225 Düsseldorf, Germany; Institute of General Practice, University Hospital Jena, Bachstraße 18, 07743 Jena, Germany; Department of Psychiatry and Psychotherapy, University of Bonn, Sigmund-Freud-Straße 25, 53105 Bonn, Germany; Institute for Social Medicine, Occupational Health and Public Health, University of Leipzig, Semmelweisstr. 10, 04103 Leipzig, Germany; Department of Health Economics and Health Services Research, University Medical Center Hamburg-Eppendorf, Martinistr. 52, 20246 Hamburg, Germany; Institute for General Practice, University of Frankfurt am Main, Theodor-Stern-Kai 7, 60590 Frankfurt am Main, Germany; Department of Medical Biometry and Epidemiology, University Medical Center Hamburg-Eppendorf, Martinistr. 52, 20246 Hamburg, Germany; Central Institute of Mental Health, Medical Faculty Mannheim/Heidelberg University, J 5, 68159 Mannheim, Germany; Institute for General Practice, WG Medical Statistics and IT-Infrastructure, Hannover Medical School, 30623 Hannover, Germany; Department of Primary Medical Care, University Medical Center Hamburg-Eppendorf, Martinistr. 52, 20246 Hamburg, Germany

**Keywords:** Socioeconomic status, Patient-reported outcomes, Multimorbid patients, Burden of disease

## Abstract

**Introduction:**

In this article three research questions are addressed: (1) Is there an association between socioeconomic status (SES) and patient-reported outcomes in a cohort of multimorbid patients? (2) Does the association vary according to SES indicator used (income, education, occupational position)? (3) Can the association between SES and patient-reported outcomes (self-rated health, health-related quality of life and functional status) be (partly) explained by burden of disease?

**Methods:**

Analyses are based on the MultiCare Cohort Study, a German multicentre, prospective, observational cohort study of multimorbid patients from general practice. We analysed baseline data and data from the first follow-up after 15 months (N = 2,729). To assess burden of disease we used the patients’ morbidity data from standardized general practitioner (GP) interviews based on a list of 46 groups of chronic conditions including the GP’s severity rating of each chronic condition ranging from marginal to very severe.

**Results:**

In the cross-sectional analyses SES was significantly associated with the patient-reported outcomes at baseline. Associations with income were more consistent and stronger than with education and occupational position. Associations were partly explained (17% to 44%) by burden of disease. In the longitudinal analyses only income (but not education and occupational position) was significantly related to the patient-reported outcomes at follow-up. Associations between income and the outcomes were reduced by 18% to 27% after adjustment for burden of disease.

**Conclusions:**

Results indicate social inequalities in self-rated health, functional status and health related quality of life among older multimorbid patients. As associations with education and occupational position were inconsistent, these inequalities were mainly due to income. Inequalities were partly explained by burden of disease. However, even among patients with a similar disease burden, those with a low income were worse off in terms of the three patient-reported outcomes under study.

**Electronic supplementary material:**

The online version of this article (doi:10.1186/s12939-015-0142-6) contains supplementary material, which is available to authorized users.

## Introduction

After having neglected older ages in the discussion about health inequalities for a long time, a growing number of studies in the recent past address health inequalities among older people. These studies indicate that there is a social gradient in morbidity and mortality among the aged, i.e. older people in a low socio-economic position generally have a higher morbidity and mortality [1-4]. Overall these inequalities tend to be smaller than among middle-age groups [4,5]. However, studies on age-related changes in health inequalities are inconsistent [3].

Although many older people suffer from more than one chronic disease, only few studies have investigated social inequalities in multimorbidity among older people. Multimorbidity can be defined as the presence of several chronic diseases in one person for a longer period of time [6]. There are three major operational definitions of multimorbidity [7]: (1) number of concurrent diseases in the same individual, (2) cumulative indices evaluating both number and severity of concurrent diseases and, (3) the simultaneous presence of diseases/symptoms, cognitive and physical functional limitations. It is known that multimorbidity has a strong impact on the affected people, including decline in functional status, lower quality of life, higher risk for mortality and increased health care utilization [7,8].

In a systematic review Marengoni et al. [7] found four cross-sectional studies reporting significant associations between low socioeconomic status (SES) and increased prevalence of multimorbidity in the elderly. Moreover, they found two prospective studies in which a low SES turned out to be a risk factor for multimorbidity incidence. Recent results of the German MultiCare Cohort Study confirmed social inequalities in multimorbidity [9]. Barnett et al. [10] examined the distribution of multimorbidity, and of comorbidity of physical and mental health disorders, in relation to age and socioeconomic deprivation in Scotland. They found that onset of multimorbidity occurred 10 to 15 years earlier in people living in the most deprived areas compared with the most affluent, with socioeconomic deprivation being particularly associated with multimorbidity that included mental health disorders. In summary, there are a few studies from different countries indicating social inequalities in multimorbidity among older persons.

Patient-reported outcomes like self-rated health, quality of life or functional limitations are considered consequences of multimorbidity [7,11]. It is known that older individuals with a low SES have a poorer self-rated health [12], poorer quality of life [13] and more functional limitations [2]. However, most of these studies did not look at the specific situation of multimorbid patients. Thus, it is fairly unclear whether deprived individuals with a similar level of multimorbidity achieve poorer outcomes [11,14].

Against this background, the analyses will address the following research questions: (1) Is there an association between SES and patient-reported outcomes in a cohort of older multimorbid patients? (2) Does the association vary according to SES indicator used (income, education, former occupational position)? (3) Can the association between SES and patient-reported outcomes be (partly) explained by burden of disease? Because differences by SES in patient-reported outcomes might have cumulated over the life course we will present both, cross-sectional analyses showing the association at baseline and longitudinal analyses of the change between baseline and follow-up after 15 months showing the ongoing development of patient-reported outcomes by SES.

## Methods

### Study design and sample

The methods of the MultiCare Cohort Study have been described in detail in the published study protocol (Trial registration ISRCTN89818205) [15]. In short, the study is designed as a multicentre, prospective, observational cohort study of multimorbid patients from general practice. The analyses presented here are based on baseline data and data from the first follow-up after 15 months. The patients were recruited from 158 general practitioner (GP) practices in 8 major cities distributed across Germany (Bonn, Düsseldorf, Frankfurt/Main, Hamburg, Jena, Leipzig, Mannheim and Munich). In each practice we created a list of patients based on the electronic database of the GP. This list encompassed all patients who were born between 1.7.1923 and 30.6.1943 (i.e. between 65 and 85 years old) and consulted the GP at least once within the last completed quarter (i.e. 3 month period). From this list we randomly selected 50 eligible patients with multimorbidity and contacted them for written informed consent. Multimorbidity was defined as coexistence of at least three chronic conditions out of a list of 29 diseases [15].

Patients were excluded from the study if they were no regular patients of the participating practice (i.e. in case of accidental consultation of the GP), if they were unable to participate in interviews (especially in case of blindness and deafness) or if they were not able to speak or read German. Further exclusion criteria were residence in a nursing home, severe illness probably lethal within three months according to the GP, insufficient ability to consent (especially dementia) and participation in other studies at the present time.

**Figure 1:**
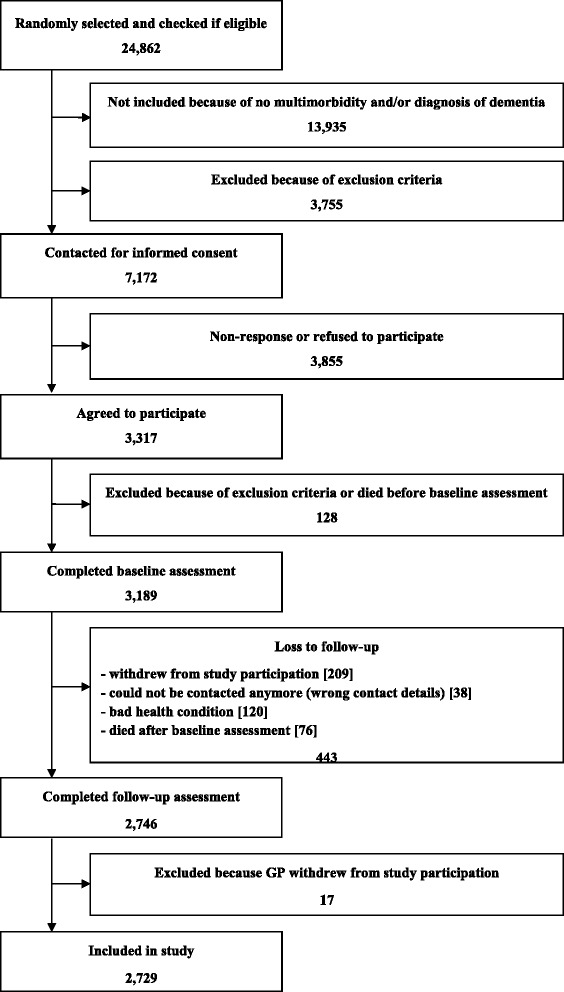
**Sampling procedure.**

Sampling procedure is shown in Figure 1. 24,862 patients were randomly selected from the study practices and checked for multimorbidity and exclusion criteria. 7,172 of these patients were eligible for study participation and contacted for informed consent to participation in our study. From all contacted patients a total of 3,855 did not participate in our study, because they refused to participate, they gave no reply, we could not obtain a valid postal address or they first agreed to participate, but it was not possible to conduct the baseline patient interview within a time frame of 16 months. 3,317 patients agreed to participate which corresponds to a total response rate of 46.2%. Retrospectively we had to exclude 128 patients, because they died before the baseline interview or we found out in contact with the patients that they complied with the exclusion criteria without the GP’s knowledge. After all, 3,189 patients could be included in the study. In terms of the follow-up after 15 months, a total of 443 of the participating patients dropped out after the baseline interview. 209 of them withdrew from study participation, because they did not want to be interviewed any more. 38 patients could not be contacted anymore, e.g. because they moved to another town. 120 patients dropped out because of bad health condition and 76 patients died after the baseline interview. In total, 2,746 patients (86.1%) completed the follow-up assessment. Another 17 patients were excluded because their GPs dropped out of the study. The final sample size for all analyses presented here was 2,729 patients. Recruitment and baseline data collection took place from July 2008 to October 2009. The follow-up was conducted between November 2009 and February 2011. The study was approved by the Ethics Committee of the Medical Association of Hamburg in February 2008 and amended in November 2008 (Approval-No. 2881).

### Measures

A comprehensive description of data sources and collected data can be found in the study protocol [15]. For the manuscript in hand we used the patients’ morbidity data from standardized GP interviews at baseline based on a list of 46 groups of chronic conditions including the GP’s severity rating of each chronic condition from this list ranging from 0 = marginal to 4 = very severe. We also included the patients’ age and gender from GP charts as well as indicators of SES, which were assessed at baseline, and patient-reported outcomes (i.e. self-rated health, health-related quality of life and functional status) from comprehensive standardized patient interviews at baseline and at follow-up after 15 months. GP interviews were conducted face-to-face in the GP practices and patient interviews were conducted face-to-face in the patients’ homes. In both cases data collection was performed by specially trained and monitored study nurses.

The methods for compiling the list of 46 diagnosis groups have been described elsewhere in detail [16]. In short, we used the most frequent conditions in GP practices as mentioned in a panel survey of the Central Research Institute of Statutory Ambulatory Health Care in Germany (“ADT-Panel”). Chronicity of diagnoses was assessed using the scientific expert report for the formation of a morbidity orientated risk adjustment scheme in the German Statutory Health Insurance. In order to capture a picture of the disease patterns in individual patients we amended this list for all chronic conditions with a prevalence ≥ 1% in the age group ≥ 65 years in the data set of the nationwide statutory health insurance company Gmünder ErsatzKasse (GEK) in 2006. For the list of diagnoses ICD-10 codes were grouped together if diseases and syndromes had a close pathophysiological similarity and if ICD codes of related disorders were used ambiguously by coding physicians in clinical reality, respectively [17].

The SES of the patients (i.e. education, income and former occupation) was assessed with a well-established standardized questionnaire [18]. The highest education grade was described according to the international CASMIN classification in nine hierarchical groups [19], which are presented in Table 1. The former occupation was grouped in five hierarchical categories according to the degree of autonomy of work [20]. Income was reported as household-size adjusted net income per month, which is calculated as household total net income per month divided by the equivalized household size, which gives 1.0 to the householder, 0.5 to other household members aged 15 or over and 0.3 to each child aged less than 15 years old [18]. Although education, income and occupation are interrelated, it is expected that associations with patient-reported outcomes vary according to the different SES indicators.

**Table 1:** **Sociodemographic and socioeconomic characteristics at baseline** (**n** = **2**,**729**)

Gender (% females)	59.1
Age (in years: mean ± sd)	74.2 ± 5.2
Education (%):	
- inadequately completed general education	1.3
- general elementary education	13.6
- basic vocational qualification or general elementary education and vocational qualification	46.0
- intermediate general qualification	2.5
- intermediate vocational or intermediate general qualification and vocational qualification	19.9
- general maturity certificate	1.1
- vocational maturity certificate/general maturity certificate and vocational qualification	4.0
- lower tertiary education	4.8
- higher tertiary education	7.0
Level of autonomy at former occupation (scaled 1–5: mean ± sd)	2.9 ± 1.2 (n = 2,713)
Net household equivalent income (in € per month: mean ± sd)	1,429 ± 719 (n = 2,424)

*n*: number of cases; *sd*: standard deviation.

We focused on the patient-reported outcomes self-rated health, health-related quality of life and functional status. Self-rated health was assessed with a visual analogue scale ranging from 0 to 100 (i.e. the best possible health status) on which the patients should mark how they did feel on the day of the interview. Health-related quality of life was assessed with the EQ-5D descriptive system [21]. The EQ-5D descriptive system includes the patients’ self-reported health state in five dimensions (mobility, self-care, usual activities, pain and anxiety/depression). From these items the EQ-5D index (i.e. a score for health-related quality of life) was calculated using the UK value set, which attaches weights that should represent the general population’s preferences to each level in each dimension and has been assessed in a population of 3.395 patients from the UK [22]. The reason for using the UK value set instead of the German value set was that the German data might be imprecise because they were based on a comparably low sample size of 380 people [22] and we assumed that there are no big cultural differences between Germany and the UK. As a further measure of functional status the instrumental activities of daily living (IADL) scale was used [23], which encompasses the items using the telephone, shopping, food preparation, housekeeping, laundry, mode of transportation, responsibility for own medication and ability to handle finances.

Missing values in the dataset arising from item non-response have been imputed in order to avoid bias generated by listwise deletion of subjects with missing values from statistical analyses [9]. We used the hot deck imputation procedure, which replaces missing values by observed values from a responding unit (donor) that is as similar as possible to the non-responding unit (recipient) regarding characteristics observed in both cases. Donors were identified as nearest neighbors based on Gower distance in a large number of auxiliary variables, which encompassed all items and scores with a proportion of missing values below 2.5%, e.g. gender, age, marital status, household type, SES indicators, morbidity, and patients’ psychosocial resources and risk factors collected in baseline interviews. A complete list of the auxiliary variables can be found in another paper [9]. A total of 2,720 patients (85.3%) were eligible as potential donors, i.e. they had complete data sets without any missing values in the auxiliary variables. We imputed missing values in the following variables: income (12.4% missing values), self-rated health (0.3%), quality of life (0.3%) and IADL (0.3%). Age, gender and education did not contain any missing values. Imputation of missing values was performed with R version 2.13.0 and the R-package StatMatch version 1.0.2.

### Analyses

We analyzed the association between SES and patient-reported outcomes cross-sectionally (i.e. at baseline) and longitudinally (i.e. the change in outcomes between baseline and follow-up after 15 months) by multilevel mixed-effects linear regression allowing for random effects at the study center and GP practice-within-study center level. All three SES indicators were introduced in the same multivariate models and each were therefore adjusted for the effect of the other indicators. In a first step, the association between SES and patient-reported outcomes was analyzed (Model 1). In a second step, burden of disease was introduced in two independent models: diseases as single variables (Model 2) and severity scores of each disease as single variables (Model 3). The degree to which the association between SES and patient-reported outcomes is explained by the two operationalizations of disease burden was assessed by the proportion to which the regression coefficient was reduced when introducing the disease burden in the models mentioned above. The percentage of reduction was calculated when the association between SES and patient-reported outcome was significant in model 1 (p ≤ 0.05). Additionally, we determined by likelihood ratio test if there was a statistically significant increase in model fit (p ≤ 0.05) from Model 1 to Model 2 and from Model 1 to Model 3, respectively. All regression analyses were controlled for age and gender and – when analyzing follow-up data – baseline-adjusted [24]. Before analysis we made a logarithmic transformation for income in order to improve the model fit. One step on the logarithmic scale equates to e.g. one of the following steps: from 400 € to 1.100 € to 3,000 € to 8,100 € net income per month. In order to make the three SES indicators comparable we reported β coefficients for one step in income (ranging from 4.2 to 9.1) or occupation (ranging from 1 to 5) but two steps in education (ranging from 1 to 9). This decision influenced the size of the β coefficients, but not the statistical significance of the analyses. The scores for IADL und quality of life were rescaled into percentages (i.e. values between 0 and 100) in order to enable a better comparison between the coefficients for all three outcome measures.

Additionally we repeated all analyses between SES and patient-reported outcomes cross-sectionally and longitudinally using statistical models in which only one single SES indicator per model was introduced. We also estimated if there were statistically significant interaction effects between age and SES indicators and between gender and SES indicators for all outcomes both cross-sectionally and longitudinally. In case of significant interaction effects we also presented subgroup analyses.

For all inferential statistics we used complete data sets including imputed data. An alpha-level of 5% (i.e. p ≤ 0.05) was defined as statistically significant. All statistical tests were conducted using Stata 11.0.

## Results

About 60% of the analyzed sample of 2,729 multimorbid patients was female (Table 1). Patients were between 65 and 85 years old, mean age was 74. SES indicators used (education, occupation and income) are shown in Table 1. About 60% of the respondents had an elementary general education which is typical for the age group under study in Germany.

The patient reported outcomes are described in Table 2. At baseline and follow-up functional status and health related quality of life had a comparable variance, but a higher mean score than self-rated health. The diseases with the highest prevalence were hypertension, lipid metabolism disorders and chronic low back pain (Table 3). The highest mean severity scores could be found in Parkinson’s disease, rheumatoid arthritis/chronic polyarthritis and obesity.

**Table 2:** **Patient**-**reported outcomes** (**n** = **2**,**729**)

	**At baseline**	**At follow** **-** **up** **(15 months)**
Self-rated health (scaled 0–100: mean ± sd)	62.9 ± 18.0	63.8 ± 18.8
	(n = 2,721)	(n = 2,721)
Functional status (IADL, scaled 0–100: mean ± sd)	79.2 ± 21.6	77.5 ± 22.7
	(n = 2,722)	(n = 2,719)
Health related quality of life (EQ-5D, scaled 0–100: mean ± sd)	80.4 ± 18.9	79.9 ± 19.6
	(n = 2,723)	(n = 2,723)

*n*: number of cases; *sd*: standard deviation.

**Table 3:** **GP**-**reported diseases and severity scores at baseline** (**n** = **2**,**729 patients**)

	**Prevalence** **(%)**	**Severity score** **(scaled 0** **–** **4:** **mean** **±** **sd)**
Hypertension	78.0	1.7 ± 0.7
Lipid metabolism disorders	59.2	1.3 ± 0.7
Chronic low back pain	49.7	2.0 ± 0.8
Joint arthrosis	44.1	2.0 ± 0.8
Diabetes mellitus	37.1	1.7 ± 0.8
Chronic ischemic heart disease	31.2	1.9 ± 0.8
Thyroid dysfunction	34.6	1.1 ± 0.7
Cardiac arrhythmias	25.7	1.6 ± 0.9
Asthma/COPD	24.1	1.8 ± 0.9
Lower limb varicosis	23.7	1.4 ± 0.8
Osteoporosis	19.8	1.7 ± 0.9
Severe vision reduction	19.2	1.5 ± 0.9
Cancers	18.1	1.4 ± 1.2
Hyperuricemia/Gout	17.2	1.0 ± 0.7
Depression	16.9	1.8 ± 0.7
Atherosclerosis/PAOD	16.4	1.7 ± 0.9
Intestinal diverticulosis	15.0	1.0 ± 0.8
Neuropathies	14.4	1.7 ± 0.8
Chronic gastritis/GERD	12.9	1.5 ± 0.7
Cardiac insufficiency	12.3	1.7 ± 0.8
Cerebral ischemia/Chronic stroke	11.8	1.6 ± 1.0
Prostatic hyperplasia	11.8	1.3 ± 0.7
Renal insufficiency	10.0	1.4 ± 0.8
Cardiac valve disorders	9.3	1.3 ± 0.9
Liver diseases	7.9	0.9 ± 0.8
Dizziness	7.8	1.6 ± 0.7
Hemorrhoids	7.7	1.1 ± 0.8
Chronic cholecystitis/Gallstones	7.7	0.7 ± 0.8
Urinary incontinence	7.2	1.7 ± 0.8
Somatoform disorders	6.1	1.8 ± 0.8
Severe hearing loss	5.4	1.9 ± 0.8
Insomnia	5.4	2.0 ± 0.8
Allergies	5.0	1.4 ± 0.9
Obesity	4.8	2.1 ± 0.9
Anxiety	4.1	1.8 ± 0.7
Rheumatoid arthritis/Chronic polyarthritis	4.1	2.1 ± 0.8
Anemias	4.0	1.2 ± 0.7
Psoriasis	3.9	1.4 ± 0.8
Migraine/chronic headache	3.6	1.4 ± 0.7
Parkinson’s disease	1.9	2.2 ± 0.9
Gynaecological problems	1.9	1.2 ± 0.7
Urinary tract calculi	1.8	1.0 ± 0.8

*sd*: standard deviation.

Cross-sectional analyses reveal that income, education and occupation were significantly associated with self-rated health at baseline, when age and gender were controlled and all SES indicators were introduced simultaneously (Model 1 in Table 4). After additional adjustment for diseases (Model 2) associations were reduced by 17% to 29%. When the disease severity score were introduced instead (Model 3), coefficients were reduced by 28% to 40%. Occupation was not significantly associated with self-rated health after adjustment for burden of disease. In terms of functional status, patients with a high income and a high occupational position rated their health significantly better. These associations were reduced by 19% to 31% but remained significant when diseases (Model 2) and disease severity (Model 3) were adjusted. Regarding health related quality of life income and education were significantly related in Model 1. Associations between education and quality of life were non-significant when burden of disease was additionally controlled.

**Table 4:** **Association between socioeconomic status** (**SES**) **and patient**-**reported outcomes** (**self**-**rated health**, **functional status and health related quality of life**) **at** ***baseline***: **multilevel mixed**-**effects linear regression**

	**Model 1**			**Model 2**				**Model 3**			
	**β**	**95%** **CI**	**p**	**β**	**95%** **CI**	**p**	**Change** ^**1**^	**β**	**95%** **CI**	**p**	**Change** ^**1**^
*Self*-*rated health*											
Income^2^	***4.34***	***2.73*** **-** ***5.95***	***<0.001***	***3.24***	***1.68*** **-** ***4.80***	***<0.001***	−25.3%*	***3.14***	***1.63***-***4.66***	***<0.001***	**−** 27.6%*
Education^3^	***1.19***	***0.42*** **-** ***1.95***	***0.002***	***0.99***	***0.25*** **-** ***1.72***	***0.009***	−16.8%*	***0.75***	***0.03***-***1.47***	***0.040***	**−** 37.0%*
Occupation^4^	***0.82***	***0.12*** **-** ***1.52***	***0.021***	0.58	−0.08-1.25	0.089	−29.3%*	0.49	−0.16-1.15	0.142	−40.2%*
*Functional status*											
Income^2^	***1.86***	***0.76*** **-** ***2.95***	***0.001***	***1.41***	***0.34*** **-** ***2.48***	***0.010***	−24.2%*	***1.29***	***0.24***-***2.33***	***0.016***	−30.6%*
Education^3^	0.04	−0.47-0.56	0.863	−0.07	−0.58-0.43	0.781		−0.13	−0.63-0.37	0.610	
Occupation^4^	***0.83***	***0.35*** **-** ***1.30***	***0.001***	***0.67***	***0.21***-***1.13***	***0.004***	−***19.3*** %*	***0.59***	***0.14***-***1.04***	***0.010***	−28.9%*
*Health related quality of life*											
Income^2^	***3.83***	***2.16*** **-** ***5.50***	***<0.001***	***2.62***	***1.02*** **-** ***4.22***	***0.001***	−31.6%*	***2.16***	***0.62***-***3.69***	***0.006***	−43.6%*
Education^3^	***0.83***	***0.03*** **-** ***1.62***	***0.041***	0.67	−0.09-0.72	0.084	−19.3%*	0.48	−0.25-1.22	0.198	−42.2%*
Occupation^4^	0.44	−0.29-1.16	0.237	0.20	−0.48-0.89	0.560		0.10	−0.56-0.76	0.772	

*Model 1*: controlled for age, gender and all other SES indicators; *Model 2*: Model 1 + diseases; *Model 3*: Model 1 + disease severity scores.

*Statistically significant (p ≤ 0.05) increase in model fit (Likelihood-ratio test) compared to Model 1.

^1^Percentage change in coefficient (Model 1 compared separately at a time with Model 2 and Model 3), percentage change is displayed when coefficient is statistically significant in Model 1 (p ≤ 0.05); ^2^β refers to one step on the logarithmic scale of the variable ranging from 4.2 to 9.1; ^3^β refers to two steps on the scale of variable ranging from 1 to 9; ^4^β refers to one step on the scale of variable ranging from 1 to 5.

95% CI*:* 95% confidence interval; significant associations (p ≤ 0.05) are italicized and bold.

In the longitudinal analyses multimorbid patients with a higher income had a significantly better self-rated health at follow-up after 15 months, controlling for self-rated health at baseline (Table 5). Education and occupation were not significantly associated with self-rated health at follow-up adjusted for baseline values (Model 1). This holds true for functional status and health related quality of life at follow-up. Associations between income and patient-reported outcomes were reduced by 18% to 27% when burden of disease was additionally adjusted (Model 2 and Model 3).

**Table 5:** **Association between socioeconomic status** (**SES**) **and change in patient**-**reported outcomes** (**self**-**rated health**, **functional status and health related quality of life**) ***between baseline and follow*** *-* ***up after 15 months***: **multilevel mixed**-**effects linear regression**

	**Model 1**			**Model 2**				**Model 3**			
	**β**	**95%** **CI**	**p**	**β**	**95%** **CI**	**p**	**Change** ^**1**^	**β**	**95%** **CI**	**p**	**Change** ^**1**^
*Self*-*rated health*											
Income^2^	***2.81***	***1.37*** **-** ***4.24***	***<0.001***	***2.18***	***0.76*** **-** ***3.59***	***0.003***	**−** 22.4%*	***2.16***	***0.76***-***3.56***	***0.003***	**−** 23.1%*
Education^3^	0.02	−0.64-0.70	0.944	0.06	−0.60-0.73	0.846		−0.08	−0.74-0.58	0.822	
Occupation^4^	0.21	−0.40-0.83	0.504	0.03	−0.57-0.64	0.911		0.05	−0.55-0.66	0.861	
*Functional status*											
Income^2^	***1.31***	***0.40*** **-** ***2.23***	***0.005***	***0.98***	***0.08*** **-** ***1.87***	***0.032***	−25.2%*	***0.96***	***0.09*** **-** ***1.84***	***0.031***	**−** 26.7%*
Education^3^	0.09	−0.33-0.52	0.671	0.13	−0.28-0.55	0.535		0.06	−0.35-0.47	0.783	
Occupation^4^	0.11	−0.58-1.00	0.598	−0.01	−0.40-0.37	0.949		0.03	−0.35-0.41	0.884	
*Health related quality of life*											
Income^2^	***2.67***	***1.24*** **-** ***4.10***	***<0.001***	***2.20***	***0.80*** **-** ***3.59***	***0.002***	**−** ***17.*** 6%*	***2.09***	***0.72*** **-** ***3.46***	***0.003***	**−** 21.7%*
Education^3^	0.13	−0.55-0.81	0.709	0.14	−0.52-0.80	0.681		0.04	−0.61-0.69	0.899	
Occupation^4^	0.26	−0.36-0.88	0.408	0.05	−0.55-0.65	0.874		0.07	−0.52-0.67	0.804	

*Model 1*: baseline-adjusted and controlled for age, gender and all other SES indicators; *Model 2*: Model 1 + diseases; *Model 3*: Model 1 + disease severity scores.

*Statistically significant (p ≤ 0.05) increase in model fit (Likelihood-ratio test) compared to Model 1.

^1^Percentage change in coefficient (Model 1 compared separately at a time with Model 2 and Model 3), percentage change is displayed when coefficient is statistically significant in Model 1 (p ≤ 0.05); ^2^β refers to one step on the logarithmic scale of the variable ranging from 4.2 to 9.1; ^3^β refers to two steps on the scale of variable ranging from 1 to 9; ^4^β refers to one step on the scale of variable ranging from 1 to 5.

95% CI: 95% confidence interval; significant associations (p ≤ 0.05) are italicized and bold.

We found a significant interaction between age and income and a significant interaction between age and occupation in the cross-sectional association with IADL, indicating that the effect of the SES indicators increases with every life year over 65, in case of income by 0.23 (p = 0.026) and in case of occupation by 0.09 (p = 0.036) percentage points (not shown). However, there were no significant interactions between age and SES indicators in the other outcomes or in the longitudinal analyses and there also were no significant interaction effects between gender and SES indicators in our analyses. (Additional file 1: Table AS1 ) shows the association between SES indicators and functional status at baseline stratified for the age groups 65–74 and 75–84. The results show that in the older age group the effects of income and occupation were increased compared to the complete sample while in the younger age group there were no significant effects of SES indicators on functional status at all.

The association between the single SES indicators and the patient reported outcomes are shown in Additional file 1: Table AS2 (cross-sectional analyses) and Additional file 1: Table AS3 (longitudinal analyses). At baseline there was a significant association between all SES indicators and all outcomes if the analyses were not adjusted for the effect of the other SES indicators (Additional file 1: Table AS2). Compared to the multivariate models the analyses of single SES indicators also show that the coefficients were slightly increased and the effect of the coefficients was reduced to a slightly greater extent by introducing the disease burden into the statistical models.

## Discussion

In this study social inequalities in patient-reported outcomes among multimorbid patients were analyzed in a cross-sectional and in a longitudinal perspective. In the cross-sectional analyses SES was significantly associated with patient-reported outcomes at baseline in a German cohort of 2,729 multimorbid patients. Associations with income were more consistent and stronger than with education and occupational position. Moreover, income was stronger associated with self-rated health and health related quality of life than with functional status. Associations were partly explained by burden of disease. For assessment of burden of disease we used patients’ morbidity data from standardized GP interviews based on a list of 46 groups of chronic conditions and the GPs severity rating of each chronic condition from this list ranging from 0 = marginal to 4 = very severe. Explanatory contribution of burden of disease varied between 17% and 44% and was higher for disease severity compared to diseases.

In the longitudinal analyses only income (but not education and occupational position) was significantly related to the patient-reported outcomes at follow-up in all three calculated models. Again, associations with self-rated health and health related quality of life were stronger than with functional status. Associations between income and the outcomes were reduced by 18% to 27% after adjustment for burden of disease, again with a higher reduction when disease severity was introduced. The higher explanatory contribution of disease severity is not surprising as GPs were asked about severity only when the patient in question suffered from the respective chronic condition.

Our results together with findings from other studies indicate a ‘double burden of disease’ among older people with a low SES. First, they have a higher risk of suffering from multimorbidity [7,9,10]. Secondly, among patients with a similar burden of multimorbidity they are worse off in terms of important outcomes like functional status, health related quality of life and self-rated health. On the other hand results suggest that patients with a low SES have worse health even after controlling for burden of disease and that about 56% to 83% of the social inequalities in patient-reported outcomes among multimorbid patients are *not* due to burden of disease. Against this background, the question arises what other factors may account for these remaining inequalities. Psychosocial factors like self-efficacy, coping behavior, social contacts, social support or psychosocial stress may play a role. Also, behavioral factors (e.g. activity level, smoking or alcohol consumption) or material factors (e.g. living conditions) may act as mediators in the association between SES and patient-reported outcomes. It is well known, that psychosocial, behavioral and material factors are important to explain and understand social inequalities in health and illness e.g. [25-27]. However, to our knowledge, up to now there is no study explicitly analyzing such explanatory factors for social inequalities in patient-reported outcomes among older multimorbid patients.

In terms of variations according to the SES indicators used, associations of patient-reported outcomes with income are stronger and more consistent than with education and occupational position. In this regard, it has to be kept in mind that the three SES indicators were introduced simultaneously into our analyses, i.e. all associations presented in the Tables 4 to 5 were adjusted for the other SES indicators. In the analyses in which the SES indicators were not introduced simultaneously, education and occupational position were significantly associated with the patient-reported outcomes at least cross-sectionally in most cases (Additional file 1: Table AS2). Thus, inequalities in patient-reported outcomes among multimorbid patients were for the most part better represented by income than by education or occupation. This is remarkable as the three indicators were only moderately correlated with each other (r = .28 to r = .50). With regard to such important outcomes among older multimorbid patients, occupation and education, both established determinants of life conditions and chances, do not seem to reflect relevant aspects of social inequality that go beyond income. This finding underlines that the three SES indicators cannot be used interchangeably, as they measure different phenomena that have a different impact depending on the population and health measure under study [28].

Some methodological aspects should be taken into account when interpreting our findings. In terms of generalizability of the results, it should be considered that the MultiCare Cohort Study is focused on elderly multimorbid patients from general practice. We decided to include only patients with at least three chronic conditions. The reason for this decision was that we wanted to avoid that almost every patient in the age group 65+ was defined as multimorbid. The data from our sampling procedure shows that - despite this restriction - our definition of multimorbidity still applies to 44% of the patients in this age group. We were able to obtain a participation rate of 46%. Although this rate is similar to other studies with a comparable design [29], we cannot rule out a selection bias due to non-response. A non-responder analysis revealed that younger patients and patients with intestinal diverticulosis or psoriasis had a better chance of study participation. However, there was no selection bias due to gender and the other 27 diseases used for patient inclusion [9]. Factors that may affect the generalizability may also result from our exclusion criteria. We had to exclude patients with dementia at baseline, because of their inability to consent. We also had to exclude patients residing in a nursing home. Finally, we recruited patients only in larger German cities, so that rural areas are not covered from our study [9].

Our measure of disease burden is confined to indicators assessing the level of multimorbidity (i.e. a list of 46 diseases and severity rating of each condition according reports from GPs). Although these indicators are in line with major operational definitions of multimorbidity [7], it is doubtable that they cover all aspects of disease burden. Moreover, in the list, 46 diseases were considered, rare diseases (i.e. prevalence less than 1% in the age group under study) were not included. However, our disease list covered the patients’ morbidity for the most part as the GPs reported a mean of 7.0 conditions from this list and a mean of 2.1 conditions per patient additionally. Furthermore, 15 months follow-up is a rather short period to analyze the associations between SES and changes in patient-reported outcomes. Finally, analyses were confined to three patient-reported outcomes (self-rated health, functional status and health related quality of life).

A strength of our study relates to a high data quality that results from the fact that interviewers were regularly trained and monitored and a multitude of procedures for prevention of insufficient data quality, detection of inaccurate or incomplete data and actions to improve data quality were performed, e.g. user reliability trainings, automatic plausibility and integrity checks and data error reports to the collaborating centres. Additional strengths consist of multivariate analyses dealing with possible confounding, multilevel models allowing for cluster effects and an advanced treatment of missing values.

We chose the hot deck approach for imputation of missing values. The strengths of this approach include that it imputes real/realistic values, that it avoids strong parametric assumptions and that it can incorporate covariate information. A weakness is that it requires good matches of donors to recipients that reflect available covariate information. For this reason comparably large data sets are needed [30]. As 2,720 patients were eligible as potential donors and income had only 12% missing values, which is a comparably low percentage for this variable, it seems rather improbable that matching problems might cause biased coefficient estimation. Nevertheless, we assessed if there is bias in our data arising from the possibility that there might be no extreme values available or that duplicate outliers might be imputed. However, we do not find these problems in our data set. On the one hand the available data of net household equivalent income have a range from 64€ to 9300€ per month and therefore incorporate extreme values. On the other hand the imputed data have a range from 100€ to 3800€ per month and do not include duplicate outliers.

## Conclusions

In conclusion, our results show social inequalities in self-rated health, functional status and health related quality of life among older multimorbid patients. As effects of education and occupational position were inconsistent, these inequalities were mainly due to income. Inequalities were partly explained by burden of disease. However, even among patients with a similar disease burden, people with a low income were worse off in terms of the three patient-reported outcomes under study.

## Additional file

Additional file 1: Table AS1. Association between socioeconomic status (SES) and functional status at baseline stratified for age groups: multilevel mixed-effects linear regression. **Table AS2.** Association between single indicators of socioeconomic status (SES) and patient-reported outcomes (self-rated health, functional status and health related quality of life) at baseline: multilevel mixed-effects linear regression. **Table AS3.** Association between single indicators of socioeconomic status (SES) and the change in patient-reported outcomes (self-rated health, functional status and health related quality of life) between baseline and follow-up after 15 months: multilevel mixed-effects linear regression.
