# Supplementary material for: Social inequalities in patient-reported outcomes among older multimorbid patients – results of the MultiCare cohort study
Source: Int J Equity Health. 2015 Feb 7;14:17. doi: 10.1186/s12939-015-0142-6 (PMC4322453; doi:10.1186/s12939-015-0142-6)
Supplement: Additional file 1: Table AS1. — Association between socioeconomic status (SES) and functional status at baseline stratified for age groups: multilevel mixed-effects linear regression. Table AS2. Association between single indicators of socioeconomic status (SES) and patient-reported outcomes (self-rated health, functional status and health related quality of life) at baseline: multilevel mixed-effects linear regression. Table AS3. Association between single indicators of socioeconomic status (SES) and the change in patient-reported outcomes (self-rated health, functional status and health related quality of life) between baseline and follow-up after 15 months: multilevel mixed-effects linear regression. [file 12939_2015_142_MOESM1_ESM.docx]

Table A1: Association between socioeconomic status (SES) and **functional status at baseline stratified for age groups**: multilevel mixed-effects linear regression

|  | Model 1 | | | Model 2 | | | | Model 3 | | | |
| --- | --- | --- | --- | --- | --- | --- | --- | --- | --- | --- | --- |
|  | β | 95% CI | P | β | 95% CI | p | Change^1^ | β | 95% CI | p | Change^1^ |
| *Age group: 65 to 74* | | | | | | | | | | | |
| Income^2^ | 0.82 | -0.30-1.95 | 0.152 | 0.57 | -0.55-1.69 | 0.321 |  | 0.34 | -0.77-1.45 | 0.547 |  |
| Education^3^ | -0.00 | -0.54-0.53 | 0.987 | -0.12 | -0.65-0.43 | 0.667 |  | -0.12 | -0.64-0.20 | 0.649 |  |
| Occupation^4^ | 0.48 | -0.04-1.00 | 0.069 | 0.44 | -0.07-0.95 | 0.093 |  | 0.41 | -0.09-0.92 | 0.109 |  |
| *Age group: 75 to 84* | | | | | | | | | | | |
| Income^2^ | ***2.93*** | ***0.98-4.89*** | ***0.003*** | ***2.24*** | ***0.34-4.13*** | ***0.021*** | ***-23.5%**** | ***2.13*** | ***0.30-3.97*** | ***0.023*** | ***-27.3%**** |
| Education^3^ | 0.22 | -0.34-0.57 | 0.627 | 0.16 | -0.73-1.04 | 0.724 |  | 0.09 | -0.76-0.95 | 0.832 |  |
| Occupation^4^ | ***1.11*** | ***0.33-1.90*** | ***0.006*** | ***0.85*** | ***0.09-1.61*** | ***0.029*** | ***-23.4%**** | 0.71 | -0.03-1.45 | 0.059 | -36.0%* |

Model 1: controlled for age, gender and all other SES indicators; Model 2: Model1 + diseases; Model 3: Model 1+disease severity scores

* statistically significant (p ≤0.05) increase in model fit (Likelihood-ratio test) compared to Model 1

^1^ Percentage change in coefficient (Model 1 compared separately at a time with Model 2 and Model 3), percentage change is displayed when coefficient is statistically significant in Model 1 (p ≤0.05); ^2^ β refers to one step on the logarithmic scale of the variable ranging from 4.2 to 9.1; ^3^ β refers to two steps on the scale of variable ranging from 1 to 9; ^4^ β refers to one step on the scale of variable ranging from 1 to 5.

95% CI: 95% confidence interval; significant associations (p ≤0.05) are italicized and bold.

Table A2: Association between **single indicators of socioeconomic status** (SES) and patient-reported outcomes (self-rated health, functional status and health related quality of life) at **baseline**: multilevel mixed-effects linear regression

|  | Model 1 | | | Model 2 | | | | Model 3 | | | |
| --- | --- | --- | --- | --- | --- | --- | --- | --- | --- | --- | --- |
|  | β | 95% CI | p | β | 95% CI | p | Change^1^ | β | 95% CI | p | Change^1^ |
| *Self-rated health* | | | | | | | | | | | |
| Income^2^ | ***5.61*** | ***4.07-7.15*** | ***< 0.001*** | ***4.19*** | ***2.70-5.68*** | ***< 0.001*** | ***-25.3%**** | ***3.88*** | ***2.43-5.34*** | ***< 0.001*** | ***-30.8%**** |
| Education^3^ | ***2.08*** | ***1.41-2.76*** | ***< 0.001*** | ***1.63*** | ***0.98-2.28*** | ***< 0.001*** | ***-21.6%**** | ***1.33*** | ***0.70-1.97*** | ***< 0.001*** | ***-36.1%**** |
| Occupation^4^ | ***1.80*** | ***1.19-2.41*** | ***< 0.001*** | ***1.33*** | ***0.74-1.92*** | ***< 0.001*** | ***-26.1%**** | ***1.13*** | ***0.55-1.71*** | ***< 0.001*** | ***-37.2%**** |
| *Functional status* | | | | | | | | | | | |
| Income^2^ | ***2.47*** | ***1.43-3.51*** | ***< 0.001*** | ***1.82*** | ***0.80-2.84*** | ***< 0.001*** | ***-26.3%**** | ***1.61*** | ***0.61-2.61*** | ***0.002*** | ***-34.8%**** |
| Education^3^ | ***0.70*** | ***0.24-1.15*** | ***0.003*** | 0.44 | -0.01-0.88 | 0.053 | -37.1%* | 0.32 | -0.11-0.76 | 0.148 | -54.3%* |
| Occupation^4^ | ***1.05*** | ***0.64-1.46*** | ***< 0.001*** | ***0.79*** | ***0.39-1.19*** | ***< 0.001*** | ***-24.8%**** | ***0.67*** | ***0.28-1.07*** | ***0.001*** | ***-36.2%**** |
| *Health related*  *quality of life* | | | | | | | | | | | |
| Income^2^ | ***4.66*** | ***3.08-6.24*** | ***< 0.001*** | ***3.16*** | ***1.63-4.68*** | ***< 0.001*** | ***-32.2%**** | ***2.51*** | ***1.04-3.97*** | ***0.001*** | ***-46.2%**** |
| Education^3^ | ***1.52*** | ***0.82-2.21*** | ***< 0.001*** | ***1.08*** | ***0.41-1.75*** | ***0.002*** | ***-28.9%**** | ***0.78*** | ***0.13-1.45*** | ***0.018*** | ***-48.7%**** |
| Occupation^4^ | ***1.21*** | ***0.58-1.84*** | ***< 0.001*** | ***0.76*** | ***0.15-1.36*** | ***0.014*** | ***-37.2%**** | 0.52 | -0.06-1.11 | 0.078 | -57.0%* |

Model 1: controlled for age and gender; Model 2: Model1 + diseases; Model 3: Model 1+disease severity scores

* statistically significant (p ≤0.05) increase in model fit (Likelihood-ratio test) compared to Model 1

^1^ Percentage change in coefficient (Model 1 compared separately at a time with Model 2 and Model 3), percentage change is displayed when coefficient is statistically significant in Model 1 (p ≤0.05); ^2^ β refers to one step on the logarithmic scale of the variable ranging from 4.2 to 9.1; ^3^ β refers to two steps on the scale of variable ranging from 1 to 9; ^4^ β refers to one step on the scale of variable ranging from 1 to 5.

95% CI: 95% confidence interval; significant associations (p ≤0.05) are italicized and bold.

Table A3: Association between **single indicators of socioeconomic status** (SES) and the change in patient-reported outcomes (self-rated health, functional status and health related quality of life) **between baseline and follow-up after 15 months**: multilevel mixed-effects linear regression

|  | Model 1 | | | Model 2 | | | | Model 3 | | | |
| --- | --- | --- | --- | --- | --- | --- | --- | --- | --- | --- | --- |
|  | β | 95% CI | p | β | 95% CI | p | Change^1^ | β | 95% CI | p | Change^1^ |
| *Self-rated health* | | | | | | | | | | | |
| Income^2^ | ***2.96*** | ***1.59-4.33*** | ***< 0.001*** | ***2.24*** | ***0.89-3.59*** | ***0.001*** | ***-24.3%**** | ***2.15*** | ***0.81-3.49*** | ***0.002*** | ***-27.4%**** |
| Education^3^ | 0.44 | -0.16-1.03 | 0.150 | 0.32 | -0.27-0.91 | 0.293 |  | 0.19 | -0.40-0.78 | 0.528 |  |
| Occupation^4^ | 0.52 | -0.03-1.06 | 0.062 | 0.28 | -0.25-0.82 | 0.300 |  | 0.24 | -0.29-0.78 | 0.370 |  |
| *Functional status* | | | | | | | | | | | |
| Income^2^ | ***1.44*** | ***0.57-2.31*** | ***0.001*** | ***1.05*** | ***0.19-1.90*** | ***0.016*** | ***-27.1%**** | ***1.02*** | ***0.18-1.86*** | ***0.018*** | ***-29.2%**** |
| Education^3^ | 0.28 | -0.09-0.66 | 0.136 | 0.23 | -0.14-0.60 | 0.218 |  | 0.17 | -0.19-0.54 | 0.349 |  |
| Occupation^4^ | 0.29 | -0.06-0.63 | 0.104 | 0.15 | -0.19-0.48 | 0.395 |  | 0.15 | -0.18-0.48 | 0.365 |  |
| *Health related*  *quality of life* | | | | | | | | | | | |
| Income^2^ | ***2.93*** | ***1.56-4.29*** | ***< 0.001*** | ***2.31*** | ***0.98-3.64*** | ***0.001*** | ***-21.2%**** | ***2.16*** | ***0.86-3.47*** | ***0.001*** | ***-26.3%**** |
| Education^3^ | 0.57 | -0.03-1.16 | 0.062 | 0.41 | -0.17-0.99 | 0.170 |  | 0.31 | -0.26-0.89 | 0.287 |  |
| Occupation^4^ | ***0.61*** | ***0.06-1.15*** | ***0.029*** | 0.33 | -0.19-0.86 | 0.214 | -45.9%* | 0.31 | -0.21-0.83 | 0.245 | -49.2%* |

Model 1: baseline-adjusted and controlled for age and gender; Model 2: Model1 + diseases; Model 3: Model 1 + disease severity scores

* statistically significant (p ≤0.05) increase in model fit (Likelihood-ratio test) compared to Model 1

^1^ Percentage change in coefficient (Model 1 compared separately at a time with Model 2 and Model 3), percentage change is displayed when coefficient is statistically significant in Model 1 (p ≤0.05) ; ^2^ β refers to one step on the logarithmic scale of the variable ranging from 4.2 to 9.1; ^3^ β refers to two steps on the scale of variable ranging from 1 to 9; ^4^ β refers to one step on the scale of variable ranging from 1 to 5.

95% CI: 95% confidence interval; significant associations (p ≤0.05) are italicized and bold.
